# FF‐QuantSC: accurate quantification of fetal fraction by a neural network model

**DOI:** 10.1002/mgg3.1232

**Published:** 2020-04-13

**Authors:** Yuying Yuan, Xianghua Chai, Na Liu, Bida Gu, Shengting Li, Ya Gao, Lijun Zhou, Qiang Liu, Fan Yang, Jingjuan Liu, Jiao Qiu, Jinjin Zhang, Yumei Hou, Miaolan Cen, Zhongming Tian, Weijiang Tang, Hongyun Zhang, Fang Chen, Ye Yin, Wei Wang

**Affiliations:** ^1^ Clinical laboratory of BGI Health BGI‐Shenzhen Shenzhen China; ^2^ BGI Genomics BGI‐Shenzhen Shenzhen China; ^3^ MGI BGI‐Shenzhen Shenzhen China; ^4^ BGI‐Shenzhen Shenzhen China; ^5^ China National GeneBank BGI‐Shenzhen Shenzhen China; ^6^ Tianjin Medical Laboratory BGI‐Tianjin BGI‐Shenzhen Tianjin China; ^7^ BGI‐Wuhan Clinical Laboratories BGI‐Shenzhen Wuhan China

**Keywords:** female pregnancy, fetal fraction, FF‐QuantSC, neural network, NIPT, shallow‐coverage sequencing

## Abstract

**Background:**

Noninvasive prenatal testing (NIPT) is one of the most commonly employed clinical measures for screening of fetal aneuploidy. Fetal Fraction (*ff*) has been demonstrated to be one of the key factors affecting the performance of NIPT. Accurate quantification of *ff* plays vital role in NIPT.

**Methods:**

In this study, we present a new approach, the accurate Quantification of Fetal Fraction with Shallow‐Coverage sequencing of maternal plasma DNA (FF‐QuantSC), for the estimation of *ff* in NIPT. The method employs neural network model and utilizes differential genomic patterns between fetal and maternal genomes to quantify *ff*.

**Results:**

Our results show that the predicted *ff* by FF‐QuantSC exhibit high correlation with the Y chromosome–based method on male pregnancies, and achieves the highest accuracy compared with other *ff* estimation approaches. We also demonstrate that the model generates statistically similar results on both male and female pregnancies.

**Conclusion:**

FF‐QuantSC achieves high accuracy in *ff* quantification. The method is suitable for application in both male and female pregnancies. Since the method does not require additional information upon NIPT routines, it can be easily incorporated into current NIPT settings without causing extra costs. We believe that FF‐QuantSC shall provide valuable additions to NIPT.

## Graphical abstract

Differential distribution between maternal and fetal genomes, potential information to be exploited by a neural network model for the prediction of fetal fraction.

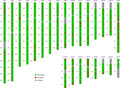

## 1 INTRODUCTION

In late 20th century, use of plasma DNA in molecular diagnosis has been demonstrated as a valuable potential (Sorenson, Pribish, Valone, & Memoli, 1994; D. Lo et al., 1997). One of the interests led to the discovery of the presence of fetal circulating cell‐free DNA (ccfDNA) in maternal plasma during pregnancy (D. Lo et al., 1997), which revolutionized the clinical application of noninvasive prenatal testing (NIPT) of fetal chromosomal aneuploidies and microdeletion/microduplication syndromes. Statistics shows that chromosomal abnormalities occur approximately 1 in 150 live births and Down syndrome (trisomy 21) occurs approximately 1 in 800 live births (Gregg et al., 2016; Mercer, 2016). The risk even increases as the mother ages (Mercer, 2016). As a result, screening of common fetal chromosomal abnormalities during pregnancy has been required by governments in many countries. The proportion of fetal ccfDNA in maternal plasma, known as the Fetal Fraction (*ff*), has been shown to be one of the fundamental factors affecting the performance of NIPT (Canick, Palomaki, Kloza, Lambert‐Messerlian, & Haddow, 2013). Previous study has revealed that *ff* is closely related to maternal weight and gestational age (Kinnings et al., 2015; Wang et al., 2013; Wataganara, Peter, Messerlian, Borgatta, & Bianchi, 2004), and the value of *ff* typically ranges from 3% to over 30% throughout the pregnancy (Canick et al., 2013). Our previous study concluded that *ff* should attain 3.5% for a reliable NIPT performance, and proceeding of NIPT with lower *ff* was associated with elevated false‐positive and false‐negative rates (Zhang et al., 2015).

*ff* may be estimated by contrasting various differential patterns between the maternal genome and the fetal genome, such as the differential methylation pattern of specific epigenetic markers (Nygren et al., 2010), the differential genotype of selected SNPs (Sparks, Struble, Wang, Song, & Oliphant, 2012; Y. M. D. Lo et al., 2010; Zimmermann et al., 2012), the size differences between fetal and maternal DNA fragments (Yu et al., 2014), and the relative coverage of the Y chromosome in male pregnancies (Lun et al., 2008). Each of these approaches has limitations yet to be overcome. The methylation‐based method requires whole‐genome bisulfite sequencing and the size‐based method requires paired‐end next‐generation sequencing. Both sequencing requirements add further cost to routine NIPT. The SNP‐based method, although accurate, utilizes extra parental SNP information, which may not be readily available. Finally, the Y chromosome method is not applicable for female fetuses.

In this report, we present a new *ff* estimation method to circumvent these limitations. The method, named the accurate Quantification of *ff* with Shallow‐Coverage sequencing of maternal plasma DNA (FF‐QuantSC), employs an artificial neural network model. It is developed upon a two‐step hypothesis: the distributions of sequencing data between maternal and fetal ccfDNA are different, and the difference is related to *ff*. While evidence from previous study provides support to the hypothesis (Kim et al., 2015), we here demonstrate a further prove. Our result shows that FF‐QuantSC achieves accurate and cost‐effective estimation of *ff* on routine NIPT settings. More importantly, FF‐QuantSC does not rely on information from fetal Y chromosome and thus also permits estimation of *ff* of female fetuses.

## 2 MATERIALS AND METHODS

### 2.1 Editorial policies and ethical considerations

This study was approved by BGI‐IRB (BGI’s institutional review board on bioethics and biosafety) and the sequencing data of all samples were obtained under BGI‐IRB approved protocols BGI‐IRB 17,048 on the BGISEQ‐1000 platform.

The pregnant women, whose samples used in this study, all signed the informed consents before sampling and agreed that the sequencing data could be used for research after anonymization. All women were of 12–24 weeks ( 17.62±4.07 ) after gestation at the time of sample collection. Gender information of fetuses was obtained by following‐up visit of testing subjects.

### 2.2 Training data and testing data

**Figure 1:**
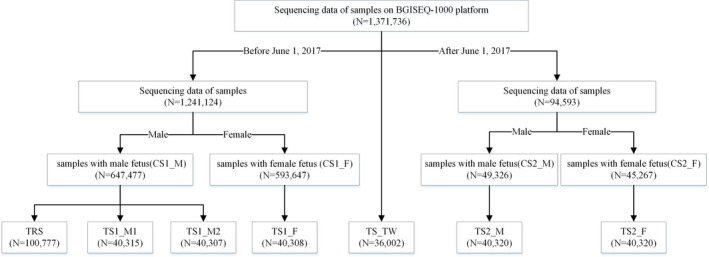
Flowchart of training set and testing set partition. Samples of singleton pregnancies were first partitioned by the time of NIPT analysis and further divided by gender. Training and testing sets were then randomly selected from each group as indicated. Samples of twin pregnancies were selected independently from the original data pool

Degree of freedom of the neural network model was calculated by the Vapnik–Chervonenkis (VC) dimension (Cherkassky, 1997). Since a rule of thumb suggested an optimal training data set 10 times of the model degree of freedom, we randomly selected 100,777 samples tested before June 1, 2017 with male fetuses as the training set (TRS). To test the robustness of FF‐QuantSC, we designed six testing sets and performed individual model testing on each set. Two testing sets (TS1_M1 and TS1_M2) were randomly selected from the remaining samples with male fetuses that were tested before June 1, 2017. A set containing female pregnancies tested before June 1, 2017 was also selected as TS1_F. Another two testing sets (TS2_M and TS2_F) were randomly generated from male and female pregnancies tested after June 1, 2017. A last set of twin pregnancies was randomly selected regardless of the testing time. Each testing set contains approximately 40,000 samples (Figure 1).

### 2.3 Sequencing data distribution between maternal and fetal ccfDNA

To confirm the two‐step hypothesis, we conducted sequencing of plasma samples from 24 randomly selected male pregnancies with a conventional protocol and a fetal‐fraction enriched protocol in parallel. The conventional procedures included plasma separation, DNA extraction, sequencing library construction, library quality control, and sequencing. Based on Lo and his colleagues’ work (Y. M. D. Lo et al., 2010), fetal‐fraction enrichment was achieved by segment selection of DNA fragments of 100–150 bp in length. A median of 8.25 (3.41–12.3) million uniquely aligned sequencing reads were obtained for each sample for subsequent analysis. These reads were partitioned into genomic regions with 60 kb windows according to their mapping positions, from which the total number of mapped reads within each window was calculated. T‐test was then applied onto each window to identify differences in read counts between the two groups of samples.

### 2.4 General flow of FF‐QuantSC

**Figure 2:**
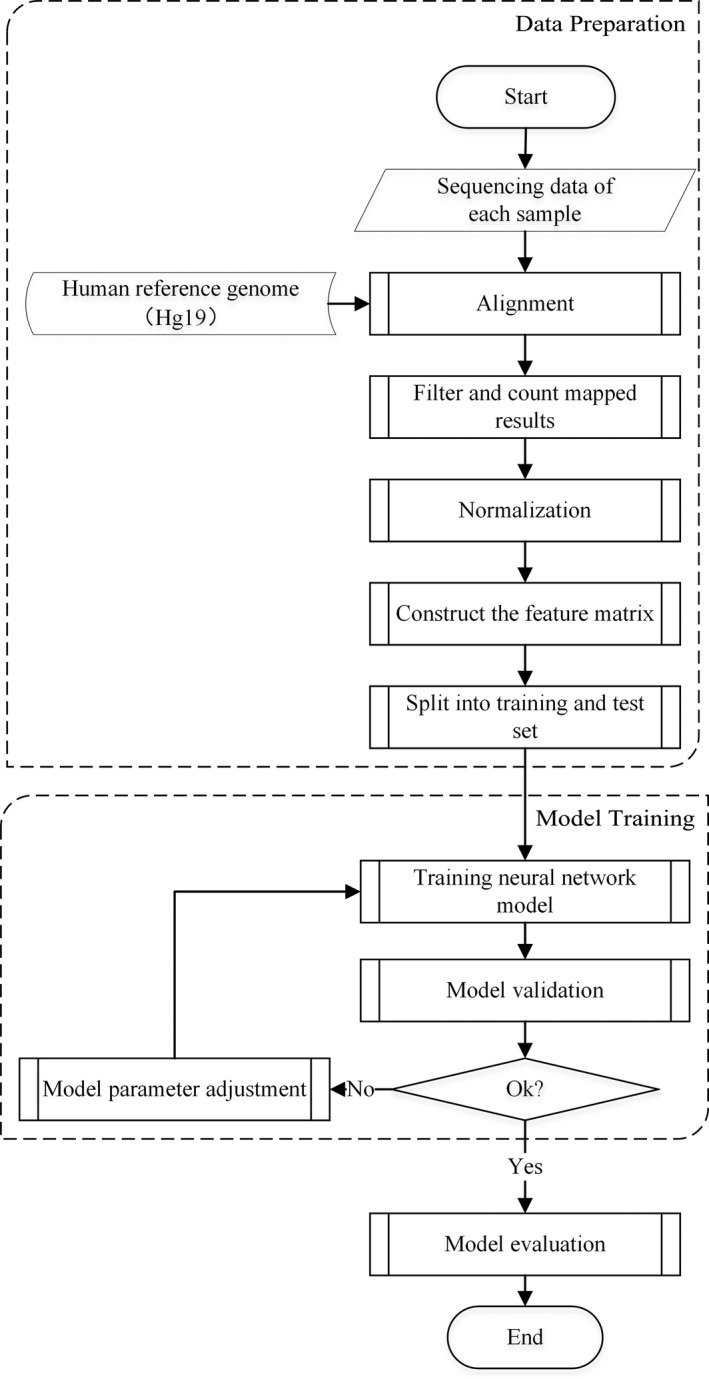
Flowchart of FF‐QuantSC method. (1) Data preparation. Sequencing reads were mapped to reference sequence (GRCh37). Mapped reads were then filtered and counted to construct feature matrixes of different datasets. (2) Model training. The network model was trained by planning procedures. (3) Model evaluation. After model training, predictive results of all test datasets were assessed to evaluate the performance of FF‐QuantSC

The overall analysis flow of FF‐QuantSC is summarized in Figure 2. All DNA sequencing was conducted on BGISEQ‐1000 platform to produce 28bp singled‐end reads. Raw sequencing data were aligned to the human reference genome (GRCh37) by BWA (V0.7.7‐r441) (Li & Durbin, 2010). Reads mapped with mismatches or more than one hit were removed. The retained effective reads were partitioned into continuous genomic windows of 60kb in length. We removed windows constantly showing no coverage and applied PCA for feature selection. Normalization was performed by dividing data of each selected feature by the sum of data of all features. The resulted data were further standardized by within‐sample z‐score transformation. This generated a final feature matrix, with each row representing a sample and each column representing a selected feature. Finally, testing results from all six testing sets were compared to evaluate the predictive capability of the trained model.

### 2.5 Model training

By comparing the fitting capability of different network structures on the training dataset, a fully connected neural network with a single hidden layer and 128 neurons was selected. Several model parameters were to be finely tuned for various purposes. The batch size was empirically set to be identical as the number of Graphics Processing Unit (GPU) cores to give the best GPU accelerating performance. Learning rate of approaches for gradient diffusion elimination was set to achieve fast model convergence. Finally, in order to balance the fitting and generalization capabilities as well as to improve the predictive accuracy of the network, two penalty factors (Goeman, 2010) and the dropout rate were autotuned by *Hyperopt* (Bergstra, Yamins, & Cox, 2013). Please refer to S1 Text and Figure S1 for more details.

## 3 RESULTS

### 3.1 Sequencing data distribution between maternal and fetal ccfDNA

**Figure 3:**
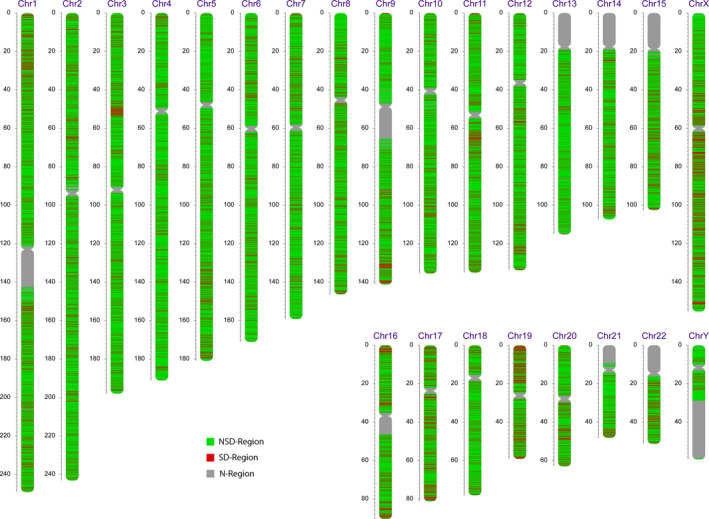
Distributional difference between maternal and fetal genomes. Red and green labels indicate regions of difference and no difference, respectively. Grey labels stand for gaps in human genome

Differential analysis of the 24 samples was summarized in Figure 3. Among all sequenced genomic regions, 8,107 of 46,981 windows were tested to show significant difference (*p* < .05) in sequencing read counts between the untreated ccfDNA samples and the fetal‐fraction enriched ccfDNA samples. This makes up roughly 17% of the entire genome (N‐regions excluded). Interestingly, instead of a sparsely random spread, these windows clustered into small groups containing up to dozens of continuous or extremely close windows, resulting in notable differential regions (*SD*‐Region) on the genome. As contrasted in red from the insignificant regions (NSD‐Region) in the figure, most of these differential regions were evenly distributed throughout the genome. However, we also noted that certain chromosomal regions harvested a higher density of the differences, such as 3p, 9q, 11q, and 19p. Since the enrichment protocol was purposed to increase the proportion of fetal ccfDNA, the observed difference should be largely attributed to elevated *ff*. This confirmed our hypothesis that there is a noticeable difference in the distribution of sequencing data between maternal and fetal ccfDNA, and this difference is related to *ff*. In turn, these differential features should provide insight for *ff* estimation in the neural network.

### 3.2 Estimation of *ff* using FF‐QuantSC

**Figure 4:**
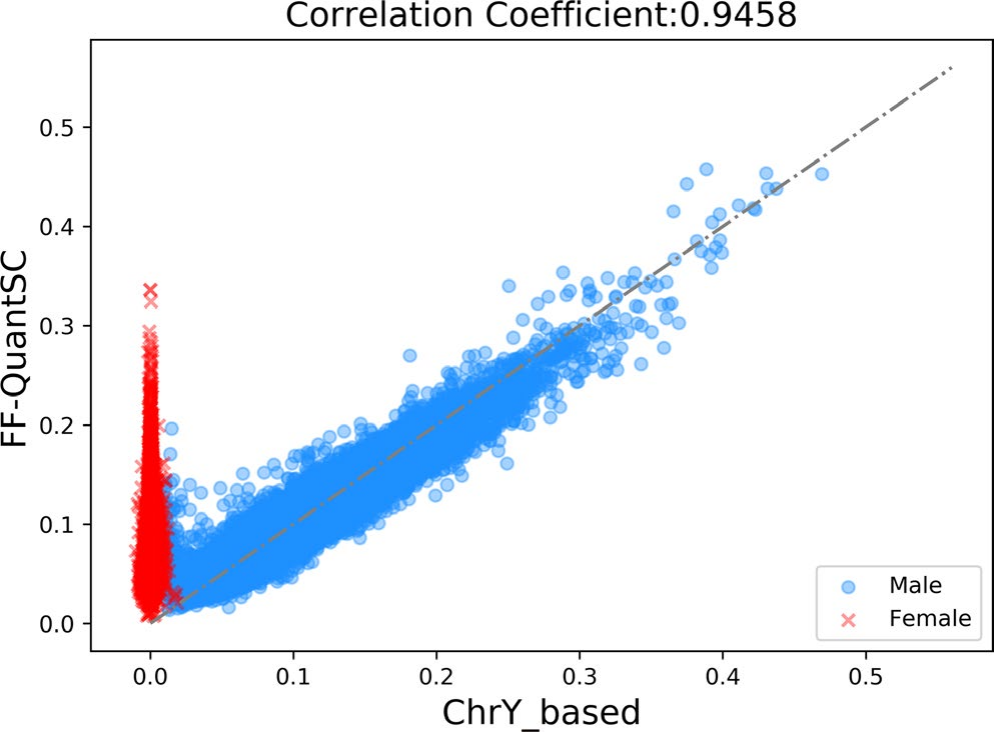
Estimated *ff* of FF‐QuantSC and Chromosome Y‐based method. X‐axis represents Chromosome Y–based *ff* estimation. Y‐axis represents FF‐QuantSC *ff* estimation. Samples with male fetuses (TS1_M1 set) are plotted by blue round dots and samples with female fetuses (TS1_F set) are plotted by red crosses. Dashed line stands for the expected correlation to be achieved

**Figure 5:**
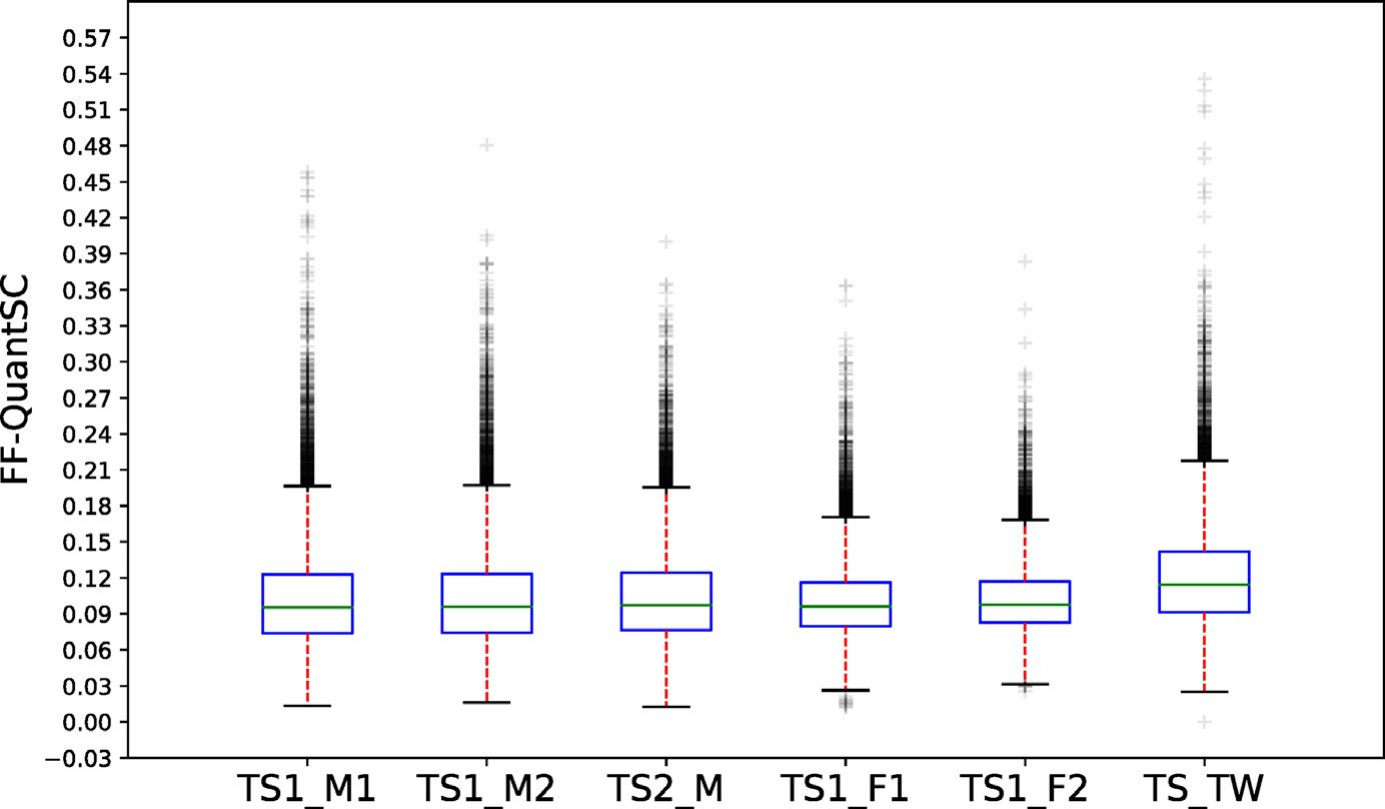
Distribution of FF‐QuantSC *ff* estimation of all testing sets. X‐axis represents test sets. TS1_M1, TS1_M2, TS2_M, TS1_F, and TS2_F are sets of singleton pregnancies and TS_TW contains twin pregnancies. Y‐axis represents FF‐QuantSC *ff* estimation. Green lines indicate the medians of predicted *ffs*

Following the confirmation of our two‐step hypothesis, we constructed and tested the neural network model. Predicted *ff* from the neural network model was compared with that from the Y chromosomal‐based method (Figure 4). As shown by male fetal samples (TS1_M1 set), *ff* results from the two methods highly correlated with each other, with a Pearson's Correlation Coefficient (PCC) of 0.9458 (*p* < .0001). We also compared the performance of FF‐QuantSC with reported performance of some of the other previously described *ff* estimation techniques (Table 1). Among all compared methods, FF‐QuantSC showed highest correlation with the Y chromosomal–based method. We thus concluded that FF‐QuantSC was able to estimate *ff* of male pregnancies with high accuracy. As also shown in Figure 4, *ff* of female fetuses (TS1_F set) estimated by FF‐QuantSC exhibited similar distribution to that of male fetuses. This suggested that it may also be feasible to apply FF‐QuantSC on female fetuses. To further demonstrate this feasibility, we compared distribution of predicted *ff* of various testing sets by FF‐QuantSC (Figure 5). As expected, among all five testing sets with singleton pregnancies, regardless of the gender of the fetus, no statistically significant differences (*p* < .0001) was detected in the distributions of estimated *ff*. This confirmed similar finding claimed by a previous study that exploited SNP‐based method (Kim et al., 2015) and suggested that FF‐QuantSC was robust and should provide equally accurate estimation of *ff* of both male and female fetuses. Finally, it is also worth noting that the twin pregnancies (TS_TW set) showed an overall elevation of the value of estimated *ff* than singleton pregnancies.

**Table 1:** Comparison of FF‐QuantSC with reported methods

Methods	PCC with Y chr‐based method	*P* value
FF‐QuantSC	0.945	<.0001
Size‐based method (Yu et al., 2014)	0.827	<.0001
SNP‐based method (Liao et al., 2011)	0.932	NA
Fetal Methylation Marker‐Based method (Chan et al., 2006; Lun et al., 2013; Nygren et al., 2010)	0.85	<.001
Cell‐Free DNA Nucleosome Track‐Based method (Straver, Oudejans, Sistermans, & Reinders, 2016)	0.636	1.61 × 10^–18^
SeqFF† (Kim et al., 2015)	0.938	NA

^†^ Note: SeqFF is a multivariate regression model and developed by Kim SK et al.

### 3.3 Comparison of FF‐QuantSC and SeqFF on Female and Twin Pregnancies

Due to lack of gold standard for *ff* estimation nonmale fetal samples, to further evaluate the performance of FF‐QuantSC, we compared FF‐QuantSC with a reasonably accurate algorithm, SeqFF, on 40,000 female and 10,326 twin pregnancies. With a PCC of 0.9201 (*p* < .0001) for female fetuses (Figure S2) and 0.8531 (*p* < .0001) for twin fetuses (Figure S3), the two algorithms showed overall high concordance.

## 4 DISCUSSION

The predicted result of TS1_M1 showed that the accuracy of FF‐QuantSC for male pregnancies was better than the other compared methods. It is worth mentioning that this is under the premise that the Y chromosomal–based method was the gold standard for the estimation of *ff* of male fetuses. Although a correlation analysis between FF‐QuantSC and SeqFF on non‐male fetuses was performed, samples with discordant *ff* estimates did not necessarily imply poor prediction by FF‐QuantSC as results from SeqFF may also be inaccurate. Distribution of the estimated *ff* by FF‐QuantSC also indicated that at extreme *ff*, error rate of *ff* prediction was much higher (Figure 4). This was possibly due to the fact that there were few samples with extreme *ff* in the training set. Therefore, FF‐QuantSC tends to learn the characteristics of samples with moderate *ff* during artificial neural network training. This provided potential direction for future model improvements. The predicted *ff* results of TS_TW showed that the median of twins is about 1.2 times that of singletons, which may also provide valuable information for future work in estimation of twin *ff*.

FF‐QuantSC requires no additional sequencing data or experimental steps to estimate *ff* in male and female pregnancies. As a result, it could be easily integrated to the routine NIPT analysis workflow without increasing the current NIPT cost. FF‐QuantSC can also be handily adjusted for different platforms. Once FF‐QuantSC is trained on a platform or sequencing mode, the model can be readily applied on any type of samples, such as pregnancies with male, female fetuses, and even potentially twins. Although high comprehensiveness and large size of the training sample are optimal in terms of model accuracy, the actual hardware support and training time should also be considered.

## 5 CONCLUSION

In this study, by comparing the performance of FF‐QuantSC with that of other *ff* estimating methods, we demonstrate that, regardless of the fetal gender, FF‐QuantSC is capable of estimating *ff* with high accuracy. The fact that FF‐QuantSC requires no additional experimental procedures on current NIPT routines enables inexpensive implementation of the model across various platforms. In summary, FF‐QuantSC shall stand as a valuable tool to the accurate estimation of *ff* in NIPT.

## 6 CONFLICT OF INTEREST STATEMENT

The authors declare no conflict of interest.

## AUTHORSHIP

Wei Wang and Yuying Yuan designed research; Xianghua Chai participated in research design, performed research, provide methodology, and software and wrote the study; Bida Gu reviewed the work, wrote the study and participated in submission; Lijun Zhou and Qiang Liu performed research and analyzed data; Miaolan Cen, Zhongming Tian, and Weijiang Tang provided data; Fan Yang, Jingjuan Liu, Jiao Qiu, Jinjin Zhang, and Yumei Hou provided validation; Ya Gao and Shengting Li reviewed the study; Na Liu, Hongyun Zhang, Fang Chen, and Ye Yin provided conceptualization.

## Supporting information

Fig S1

Fig S2

Fig S3

Supplementary Material

## Data Availability

Research data are not shared.
