# Supplementary figures and images for: FF‐QuantSC: accurate quantification of fetal fraction by a neural network model
Source: Mol Genet Genomic Med. 2020 Apr 13;8(6):e1232. doi: 10.1002/mgg3.1232 (PMC7284026; doi:10.1002/mgg3.1232)

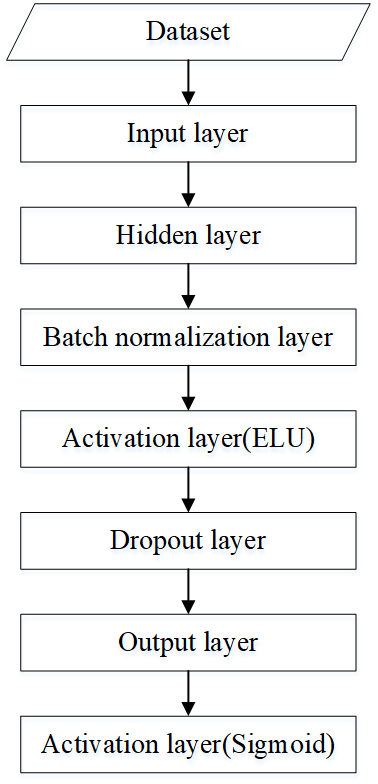

Supplement: Supplementary file 1 — Fig S1 [file MGG3-8-e1232-s001.tif]

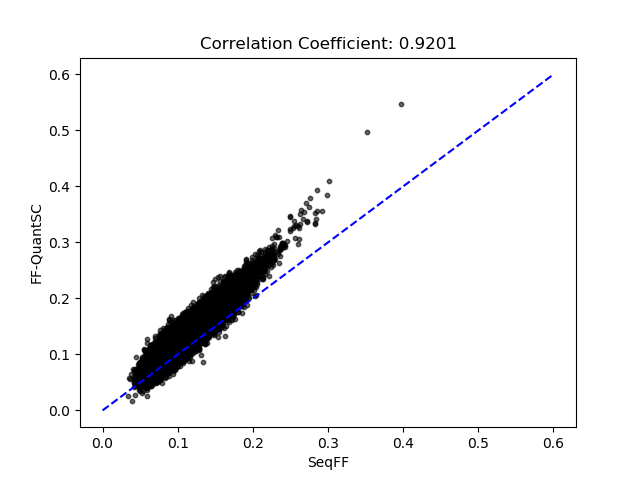

Supplement: Supplementary file 2 — Fig S2 [file MGG3-8-e1232-s002.tiff]

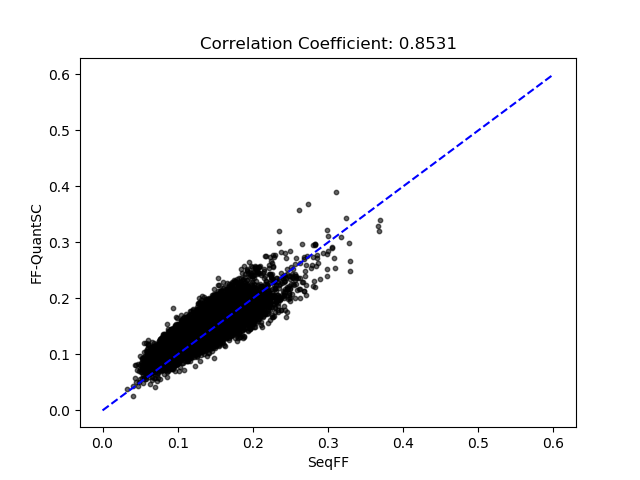

Supplement: Supplementary file 3 — Fig S3 [file MGG3-8-e1232-s003.tiff]
