# Supplementary material for: FF‐QuantSC: accurate quantification of fetal fraction by a neural network model
Source: Mol Genet Genomic Med. 2020 Apr 13;8(6):e1232. doi: 10.1002/mgg3.1232 (PMC7284026; doi:10.1002/mgg3.1232)
Supplement: Supplementary file 4 — Supplementary Material [file MGG3-8-e1232-s004.docx]

**Supporting Information**

**S1 Text**

The system environment on which model training depends was *Ubuntu 16.04.3 LTS*. The software used in model training included *Conda* (4.3.25), *Python* (3.6.2), *Keras* (2.0.8) (Chollet François, 2015), *Theano* (0.9.0) (J. Bergstra et al., 2010), *Hyperopt* (0.1) (J. S. Bergstra, Yamins, & Cox, 2013), *Numpy* (1.13.1), *Pandas* (0.20.3) (McKinney & Team, 2015) and *Scikit-learn* (0.19.0) (Pedregosa et al., 2011). Before model training, feature selection, data standardization, and model structure design were performed.

**Feature Selection.** When analyzing data with high dimensional features, it is usually advisable to perform feature selection. In this study, feature selection was performed to eliminate 1) the effect of positive samples on the predicted results; 2) problem of overfitting and a circular demonstration; 3) the collinearity between features; 4) features that are independent of predictive variables. As a result, features of chromosome 13, 18, 21, X and Y were first excluded. We then tested 3 different feature selection strategies: 1) Least Absolute Shrinkage and Selection Operator (LASSO). We carried out a 5-fold cross validation. The penalty factor was determined as 1.6e-7, and effective features were selected for the model. 2) Univariate feature selection. We calculated the correlation coefficient of each feature with the target variable. Only features that were strongly related to the target variable (*r^2^*>0.6 and *P*<0.05) were selected for the model. 3) Principal component analysis (PCA). After removing the redundant features with small eigenvalues, other features were selected. PCA was chosen as the final approach and this resulted in 4000 features for subsequent analysis.

**Data Standardization.** In order to maintain the numerical range of all characteristics at the same level, we standardized the PCA transformed features in two steps. First, data of each feature was divided by the sum of data of all features in a sample. The resulted data then underwent Z-score standardization.

**Neural Network Structure Design.** Overview of the neural network structure is summarized in **Figure S1**. The number of layers of the network and the number of neurons per layer were determined according to the principle of Occam’s Razor (Michael I. Jordan, LeCun, & Solla, n.d.). For computational convenience, we started with a single hidden layer fully-connected neural networks with $2^{n} (n is an integer)$ neurons (64, 128, 256, 512 etc.). To minimize the Mean Squared Error (MSE) between the predicted *ff* and the result estimated by chromosome Y-based method (from TS1_M1 set), while also taking the speed of network training into consideration, we selected the network with 128 hidden units. Next, we examined performance of various fully-connected neural networks with 128 hidden units but different hidden-layer structures. Results showed that no obvious improvement was made with increased network layer (**Table S1**). Eventually, we selected the model with a single hidden layer, 128 hidden units and fully-connected neural network.

**Training Strategy.** A mini-batch strategy was used to train the neural network. In each epoch, with a specified batch size, only part of the samples was used for training. The network continued to the next iteration until all samples have been adopted. In general, as batch size increases, more rapid training and enhanced model is expected. However, increased batch size also leads to weaker generalization capability of the neural network. Adam optimizer was selected for optimization, which could speed up the convergence process and save plenty of time for parameter-tuning. MSE was adopted as the loss function of the network.

**Parameter Tuning.** In neural network, large learning rate may cause skipping of the optimal point in model training and small learning rate often leads to slow convergence. Another important factor that may also impact model convergence is gradient diffusion. To minimize the time of convergence, we employed the Exponential Linear Unit (ELU) activation function to reduce the gradient diffusion in the network and adopted the Batch Normalization layers to keep the data flowing in the network under a standard normal distribution. This allowed the network weights to be updated effectively within a reasonable range and thus permitted a large learning rate. Network weights were initialized with He Normal Initializer and automatically tuned by the *Hyperopt* (J. S. Bergstra et al., 2013) package in Python. Finally, to prevent overfitting, we introduced *L1* and *L2* regularizations (Goeman, 2010) and dropout strategy. Values of these parameters were initialized to zero and the optimal values were also chosen by the *Hyperopt* (J. S. Bergstra et al., 2013) package. The tool reaches a superior hyper-parameters combination in a given high-dimensional space through either a random search strategy or a Bayesian search strategy. Please refer to **Table S2** for the list of all specifications of the final model.

**References**

Bergstra, J., Breuleux, O., Bastien, F., Lamblin, P., Pascanu, R., Desjardins, G., … Bengio, Y. (2010). Theano: a CPU and GPU Math Expression Compiler. In *SciPy*.

Bergstra, J. S., Yamins, D., & Cox, D. D. (2013). Hyperopt: A python library for optimizing the hyperparameters of machine learning algorithms. In *12th Python in science conference (SCIPY 2013)*. https://doi.org/10.1088/1749-4699/8/1/014008

Chollet François. (2015). Keras: The Python Deep Learning library. *Keras.Io*. https://doi.org/10.1086/316861

Goeman, J. J. (2010). L1 penalized estimation in the Cox proportional hazards model. *Biometrical Journal*. https://doi.org/10.1002/bimj.200900028

McKinney, W., & Team, P. D. (2015). Pandas - Powerful Python Data Analysis Toolkit. *Pandas - Powerful Python Data Analysis Toolkit*. https://doi.org/10.1073/pnas.1803154115

Michael I. Jordan, LeCun, Y., & Solla, S. A. (n.d.). Advances in Neural Information Processing Systems: Proceedings of the First 12 Conferences. In M. I. Jordan, Y. LeCun, & S. A. Solla (Eds.), *Advances in Neural Information Processing Systems: Proceedings of the First 12 Conferences* (pp. 294–300). The MIT Press.

Pedregosa, F., Varoquaux, G., Alexandre, G., Michel, V., Thirion, B., Grisel, O., … Edouard, D. (2011). Scikitlearn: Machine Learning in Python Gaël Varoquaux. *Journal of Machine Learning Research*. https://doi.org/10.1007/s13398-014-0173-7.2

**Tables**

**Table S1. Training results of different network structures**

| Network structure | | | MSE |
| --- | --- | --- | --- |
| 1^st^ hidden layer | 2^nd^ hidden layer | 3^rd^ hidden layer |  |
| 128 | N/A | N/A | 2.46e-4 |
| 64 | 2 | N/A | 2.78e-4 |
| 32 | 4 | N/A | 2.75e-4 |
| 32 | 2 | 2 | 3.19e-4 |

**Table S2. Selected network structure and parameters**

| Network structure | 1 hidden layer with 128 units |
| --- | --- |
| Learning rate | 0.001 |
| Batch size | 1920 |
| Activation | ELU |
| Weight initial method | He Normal Initializer |
| Optimizer | Adam |
| L1 norm | 1.9e-6 |
| L2 norm | 6.34e-6 |
| Dropout rate | 0.0233084 |

**Figure Legends**

**Figure S1. Conceptual diagram of network structure.** The neural network could be conceptualized as a multi-layer structure. From the input layer, a hidden layer is formed. The output layer is reached through a series of mathematical transformations. After activation, the final prediction is generated.

**Figure S2. Estimated ff of FF-QuantSC and SeqFF Method on Female Fetuses.** X-axis represents SeqFF *ff* estimation. Y-axis represents FF-QuantSC *ff* estimation. Dashed line stands for the expected correlation to be achieved.

**Figure S3. Estimated ff of FF-QuantSC and SeqFF Method on Twin Fetuses.** X-axis represents SeqFF *ff* estimation. Y-axis represents FF-QuantSC *ff* estimation. Dashed line stands for the expected correlation to be achieved.
